# Open questions on aromaticity in organometallics

**DOI:** 10.1038/s42004-020-00419-5

**Published:** 2020-11-10

**Authors:** Jun Zhu

**Affiliations:** State Key Laboratory of Physical Chemistry of Solid Surfaces and Collaborative Innovation Center of Chemistry for Energy Materials (iChEM), Fujian Provincial Key Laboratory of Theoretical and Computational Chemistry and Department of Chemistry, College of Chemistry and Chemical Engineering, Xiamen University, Xiamen, 361005 China

**Keywords:** Computational chemistry, Chemical bonding

## Abstract

While *sp*^2^-hybridized carbon atoms in hydrocarbons typically contribute only one electron to their aromaticity, metals have more electrons from *d* or *f* orbitals available for participating in conjugation in organometallics, complicating the electron counting as well as analysis of their aromaticity. Here, the author comments on the challenges towards understanding aromaticity in organometallics and outlines several remaining questions that have yet to be answered.

The concept of aromaticity, originally proposed for benzene and hydrocarbons with low C:H ratios owing to their pleasant smells (aromas), was used to explain their enhanced thermodynamic stabilities in comparison with their acyclic counterparts, caused by their resonance structures (Fig. 1a)^1,2^. Nowadays, aromaticity has been extended from organics to organometallics^3^ to inorganics^4^, and from the ground state to the excited state^5,6^, leading to various applications of aromatics in functional materials and reaction mechanisms^7,8^. In comparison with traditional organics, analyzing the aromaticity in organometallics (Fig. 1) is still challenging and several open questions are listed below to invite both experimentalists and theoreticians for their examination.

**Fig. 1:**
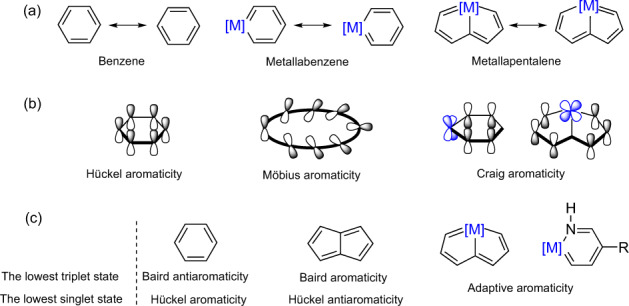
Comparison of aromaticity between organics and organometallics in both the lowest singlet and triplet states. **a** The resonance structures of benzene, metallabenzene, and metallapentalene. **b** Proposed alternative classification of aromaticity. **c** Adaptive aromaticity in organometallics in comparison with traditional Hückel and Baird aromaticity. [M] = A metal fragment; R = A phosphonium substituent.

## The nature of aromaticity in organometallics

Although various aromaticity indices have been proposed, it is still challenging to quantify aromaticity in organometallics. For instance, nucleus-independent chemical shift (NICS) values could be significantly affected by the metal centers^9^ or ligands close to a ghost atom (placed at the ring center for a σ system or 1.0 Å above or below the ring center for a π system in most cases). The harmonic oscillator model of aromaticity (HOMA) values are also unavailable in organometallics due to a lack of a parameterization for transition metal–carbon bonds^10^. Whatever method is used, trying aromaticity indices (based on different physicochemical properties) as much as possible is always recommended^11^. In many cases, the contradictions between different indices are caused by the failure of some indicators to evaluate aromaticity correctly rather than by the multidimensional character of aromaticity. On the other hand, the current classification of aromaticity in organometallics is mainly based on the topology and number of π-electrons, similar to that in organics, leading to two types of aromaticity, specifically, Hückel aromaticity (with 4*n*+2 π-electrons)^12^ and Möbius aromaticity (4*n* π-electrons)^13^. However, early in 1958, 6 years before Heilbronner proposed the concept of Möbius aromaticity^13^, Craig et al. proposed a novel type of aromaticity^14^, which was later termed as Craig-type Möbius aromaticity^15^. Note that when more rings are involved in the conjugation, exclusively classifying some metallacycles as having Hückel or Möbius aromaticity becomes difficult and even unnecessary because they have a hybrid Hückel–Möbius or even quasi-aromatic nature^16^. To give a full credit to Craig, the original proposer for 4*n* π-aromaticity, an alternative is proposed here that three rather than two types of aromaticity in organometallics could be more suitable, namely, Hückel aromaticity, Craig aromaticity and Möbius aromaticity (Fig. 1b). The first one contains 4*n*+2 π-electrons, whereas both the second and the third contain 4*n* effective π-electrons. The difference between the second and the third solely depends on the topology of a molecule. The former has a planar ring whereas the latter possesses a Möbius topology.

## Electron counting on hyperconjugative aromaticity in organometallics

The electronic configuration (ground state) of carbon is 1*s*^2^2*s*^2^2*p*^2^, whereas that of transition metals becomes (*n*-1)*d*^1:10^*ns*^1:2^ (*n* = 4,5,6). Thus, more electrons from *d* orbitals of a transition metal, in contrast to one electron from a carbon atom, can participate in the conjugation of a cyclic species, leading to a challenge in electron counting in organometallics. For instance, the number of π-electrons in a metallabenzene complex could vary from six to ten depending on metal centers, geometries and ligands^17^. In addition, the number of formal π-electrons of the first isolated metallapentalyne is ten. However, the nature of its aromaticity is eight-center-eight-electron Craig aromaticity^18^. Thus, electron counting in metallacycles is far from trivial. It is particularly challenging to determine the number of π-electrons in hyperconjugative aromaticity of organometallics as more π-electrons than six could be located in transition metal-substituted pyrrolium rings^19^.

## σ-aromaticity in unsaturated organometallics

In general, aromaticity could be classified as σ- and π-aromaticity according to the character of the cyclic electron delocalization, distributed in saturated and unsaturated systems, respectively. Crossing to the opponent’s domain seems unlikely. Interestingly, σ-aromaticity dominating in an unsaturated organometallic system has been reported recently in a metallacyclopropene unit^3^. It is still challenging to dig out σ-aromatic unsaturated organometallics, especially in other-membered rings because π-aromaticity in most cases prevails in such an unsaturated domain.

## Adaptive aromaticity in organometallics

Cyclic species can be aromatic either in the ground state or the excited state according to Hückel and Baird rules, which could be regarded as one-state aromaticity. Aromatic species that exist in two states, e.g., the lowest singlet state (S_0_) and the lowest triplet state (T_1_) seems impossible owing to a violation of Hückel and Baird rules. Very recently, our group proposed two-state aromaticity in a 16e osmapentalene (Fig. 1c), which is aromatic in both S_0_ and T_1_ states, and which we termed as adaptive aromaticity^20^. Later, the concept of adaptive aromaticity has been extended to osmapyridinium^21^, mono-substituted benzene, tetraatomic boron species, osmapentalene derivatives, cyclo[10]carbon, and the pyrrole ring in dipyrrolonaphthyridinedione^22^. As two-state aromaticity is particularly rare, discovery of such adaptive aromatics, caused by a novel excitation fashion, has been always challenging. As the nature of aromaticity in metallabenzene remains ambiguous, the unpredictability in adaptive aromaticity could become more significant.

## Aromaticity-driven dinitrogen activation in organometallics

**Fig. 2:**
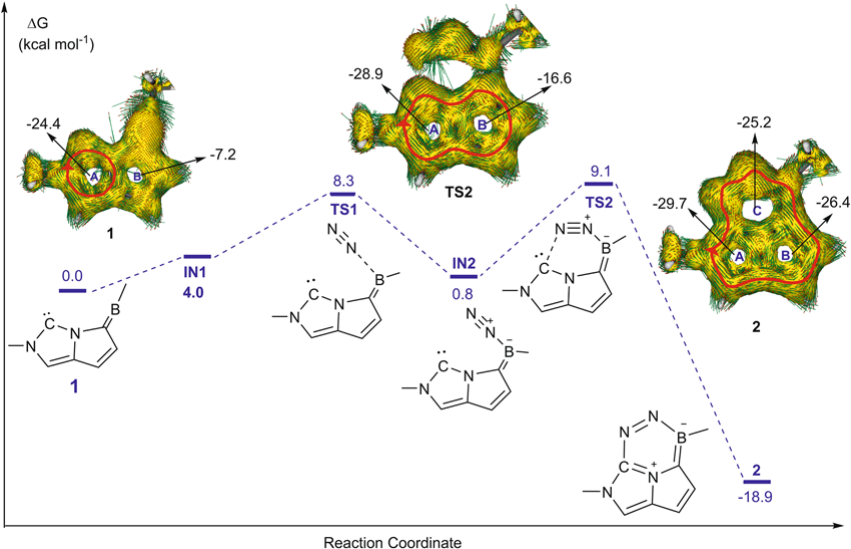
Predicted dinitrogen activation by a frustrated Lewis pair. Gibbs free energy profiles for the activation of N_2_ by carbon‐boron frustrated Lewis pair **1** with selected ACID plots with an isovalue of 0.04 a.u. and NICS(1)_*zz*_ values (ppm) of rings A, B, and C in **1**, **TS2**, and **2**. Adapted with permission from ref. ^23^. Copyright 2019 Wiley-VCH Verlag GmbH & Co.

Aromaticity could stabilize not only an intermediate or a product in a given reaction but also the transition state. Recently, we proposed a novel approach for dinitrogen activation by a frustrated Lewis pair **1** via density functional theory calculations (Fig. 2)^23^. The number of aromatic rings could be gradually increased from one (in a reactant) to two (in a transition state) to three (in a product) via a [4 + 2] cycloaddition, which is supported by the negative NICS(1)_*zz*_ values and clockwise ring currents in the anisotropy of the current-induced density (ACID) plots. Thus, dinitrogen activation becomes favorable both thermodynamically (with an exothermicity of Gibbs free energy by 18.9 kcal mol^−1^) and kinetically (with an activation energy as low as 9.1 kcal mol^−1^). Developing aromaticity-driven transition meal-involved dinitrogen activation should be challenging due to the strong triple bond of dinitrogen, but represents an example where aromaticity in organometallics could potentially be harnessed for practical applications.

## Outlook

The diversity of transition metals significantly enriches the chemistry of metalla-aromatics where a metal fragment is used to replace the CH group in an organic species. In particular, metal-bridgehead aromatics and spiro metalla-aromatics become possible as some of the organic counterparts are either particularly challenging or impossible due the limited mode of the carbon coordination^18,24^. As every coin has two sides, the more electrons from *d* or *f* orbitals definitely complicate the aromaticity in organometallics, enhancing the uncertainties of aromaticity. For instance, the aromaticity reversal between metalla-aromatics and the organic parent has been found in carbolong chemistry from Hückel aromaticity to Craig aromaticity^8^. On the other hand, in comparison with various metalla-aromatics, realizing metalla-antiaromatics is even more challenging owing to the destabilization of antiaromaticity. As most reported organometallics are transition metal involved, developing *f*-block aromatic organometallics^25^ is extremely challenging as most *f*-block elements favor a bonding with oxygen and nitrogen atoms. With the rapid development of computational power, calculations will not only contribute significantly to understanding the aromaticity of transition states as well as products in reaction mechanisms in organometallic chemistry, but also play an important role in predicting novel metalla-(anti)aromatics and their computationally screened pathways for experimental examination.
